# Diagnostic performance of endoscopic ultrasound-guided tissue acquisition by EUS-FNA versus EUS-FNB for solid pancreatic mass without ROSE: a retrospective study

**DOI:** 10.1186/s12957-022-02682-3

**Published:** 2022-06-24

**Authors:** Thanawin Wong, Tanawat Pattarapuntakul, Nisa Netinatsunton, Bancha Ovartlarnporn, Jaksin Sottisuporn, Naichaya Chamroonkul, Pimsiri Sripongpun, Sawangpong Jandee, Apichat Kaewdech, Siriboon Attasaranya, Teerha Piratvisuth

**Affiliations:** 1 Division of Gastroenterology and Hepatology, Department of Internal Medicine, Faculty of Medicine, Prince of Songkla University, Hatyai, Songkhla 90110 Thailand; 2 NKC Institute of Gastroenterology and Hepatology, Faculty of Medicine, Prince of Songkla University, Hat Yai, Songkhla Thailand

**Keywords:** Endoscopic ultrasound-guided tissue acquisitionFine-needle aspiration, Fine-needle biopsy, Solid pancreatic mass, Franseen needle, Diagnostic performance

## Abstract

**Background:**

Endoscopic ultrasound-guided tissue acquisition (EUS-TA) is an established diagnostic procedure for solid pancreatic mass. However, the diagnostic yield between fine-needle aspiration (FNA) and fine-needle biopsy (FNB) remains unclear. We aimed to evaluate and compare the diagnostic yields between FNA and FNB using conventional FNA and Franseen needles of the same size 22-gauge needle, in patients with solid pancreatic mass who underwent EUS-TA without rapid onsite cytopathology evaluation (ROSE).

**Methods:**

All cases of EUS-TA by FNA or FNB for solid pancreatic mass between January 2017 and October 2020 in a single-centre university hospital were retrospectively reviewed. All procedures were performed without an onsite cytologist. Before the endoscopist finished the procedure, macroscopic onsite evaluation (MOSE) was confirmed. The diagnostic yield and the average number of needle passes between FNB and FNA were then compared.

**Results:**

A total of 151 patients (FNA, *n* = 77; FNB, *n* = 74) with solid pancreatic mass detected by cross-sectional imaging underwent EUS-TA. The mean age was 62.3 ± 12.8 years, with 88 (58.3%) males. Age, sex, mass location, tumour size and disease stage from imaging were not significantly different between the two groups. The diagnostic performance was higher in EUS-FNB (94.6%) than in EUS-FNA (89.6%). The mean number of needle passes was clearly fewer in FNB than in FNA (2.8 vs. 3.8, *p* < 0.001). The total procedure time was shorter in FNB (34.7 min) than in FNA (41 min). The adverse event rate between FNB and FNA was not significantly different.

**Conclusions:**

The diagnostic yield of solid pancreatic mass was higher in FNB using the Franseen needle than in FNA using the conventional FNA needle in a centre where ROSE is unavailable, without serious adverse event. In addition, FNB had fewer needle passes and shorter total procedure time.

## Introduction

Pancreatic cancer is known to be associated with an unfavourable prognosis as a delayed diagnosis is commonly encountered. Symptomatic pancreatic cancer patients are usually beyond curative treatment, and despite various treatment options for patients with advanced-stage pancreatic cancer, to date, the survival outcome had not been satisfactory high; therefore, early diagnosis and treatment of pancreatic cancer are necessary for longer survival [1, 2].

Endoscopic ultrasound-guided tissue acquisition (EUS-TA) has been the main diagnostic tool for solid pancreatic mass [3, 4]. EUS-guided fine-needle aspiration (FNA) is the standard procedure for tissue sampling, with sensitivity and specificity of 64–95% and 75–100%, respectively [5–8]. Nonetheless, EUS-FNA often provides small tissue samples and inadequate specimens for a definite diagnosis. Optimal EUS-TA depends on many factors, including an endosonographer’s expertise, needle type and needle size, tissue sampling technique (stylet slow-pull or standard suction), tumour location, tumour size and rapid onsite cytopathology evaluation (ROSE) availability; in many centres, ROSE is unavailable [8]. In pancreatic adenocarcinoma, cytology is sufficient for diagnosis; meanwhile, other diseases (such as neuroendocrine tumour, lymphoma, autoimmune pancreatitis, tuberculosis and mass-forming chronic pancreatitis) require core tissue sample and immunohistochemistry staining [9]. EUS-guided fine-needle biopsy (EUS-FNB) is another procedure technique that may be better in obtaining a core tissue sample, hypothetically. However, previous studies using the first- and second-generation biopsy needles reported no superior outcomes of EUS-FNB compared with EUS-FNA using conventional FNA needle [10–12]. In recent years, tissue acquisition using the third-generation biopsy needles, e.g. forward-facing bevel needles (Procore; COOK Medical, Bloomington, Indiana, USA), fork-tip needles (SharkCore; Medtronic, Dublin, Ireland) and Franseen needles (Acquire; Boston Scientific, Marlborough, Massachusetts, USA), for solid pancreatic lesions was widely performed. This newer generation biopsy needle can obtain a larger amount of core tissue specimens which might lead to a better yield for definite diagnosis [13, 14]. The European Society of Gastrointestinal Endoscopy (ESGE) guideline recommends both 25- and 22-gauge needles for tissue sampling in solid pancreatic mass regardless of the needle type [8]. There were some studies exploring the role of 3rd-generation FNB needles. A study by Panic and Larghi showed that EUS-FNB demonstrated a better diagnostic yield than EUS-FNA, with fewer needle passes, shorter procedure time, lower adverse events and increased histopathology yield [15]. Rodrigues-Pinto et al. reported that EUS-FNB without ROSE had a numerically higher diagnostic yield for malignancy (90%) than EUS-FNA with ROSE (77.5%), yet the difference was not statistically significant [16]. Recently, Crino et al. reported that the diagnostic accuracy of EUS-FNB using new generation FNB needles without ROSE was comparable to EUS-FNB with ROSE in solid pancreatic lesions [17].

In Thailand, the limitation of EUS-TA is biopsy needle cost and the unavailability of ROSE generally. In this study, we aimed to compare the diagnostic yield between EUS-FNA and EUS-FNB utilizing the 3rd-generation biopsy needles, when the same needle size of 22 gauge was used in patients with solid pancreatic mass without ROSE. The average number of needle passes, the total procedure time and adverse events were also compared between these two groups.

## Methods

We conducted a retrospective cohort single-centre study, including patients with solid pancreatic mass who underwent EUS-TA between January 2017 and October 2020. Specifically, all procedures were performed at the Institute of Gastroenterology and Hepatology, Songklanagarind Hospital, the only university hospital in Southern Thailand. The inclusion criteria were as follows: (1) evidence of the solid pancreatic mass confirmed by computed tomography (CT) scan or magnetic resonance imaging (MRI), (2) age at least 18 years, (3) technical success of EUS-FNA or EUS-FNB without ROSE and (4) no prior EUS-TA done. Patients who were scheduled to undergo EUS-TA but with uncorrectable coagulopathy or any other reasons which caused EUS-TA to be inoperable were, therefore, excluded. We identified eligible patients according to the inclusion and exclusion criteria from our registration database, and using the hospital’s medical electronic database, the patients’ and procedural data were collected.

The study protocol was approved by the Faculty of Medicine Institutional Review Board. The informed consent for the study was waived as this is retrospective in nature. However, all patients provided informed consent for EUS-TA before the procedures started.

### Identification of solid pancreatic mass and the decision to perform EUS-TA

The identification of solid pancreatic mass on CT or MRI was certified by experienced radiologists. In our centre, the routine practice for patients with solid pancreatic mass depends on attending physicians. Usually, authorized physicians (gastroenterologists, oncologists or hepatobiliary surgeons) referred patients to advance endoscopists for tissue diagnosis before initiating systemic chemotherapy in unresectable patients or in patients with questionable diagnoses. However, the small solid pancreatic mass within resectable criteria may proceed to surgical resection without EUS-TA for tissue diagnosis.

### EUS-FNA or EUS-FNB and cyto/histopathological evaluation

In this study, EUS was performed using a linear array echoendoscope (GF-UCT/P-180 series; Olympus Medical System, CORP., Tokyo, Japan) and ultrasound machine (Aloka; model SSD alpha 10, Tokyo, Japan) under conscious sedation by four experienced endosonographers (TP, NN, JS and BO). All patients with their first-time experiences of FNA or FNB for tissue acquisition in solid pancreatic mass were included.

The general protocol for EUS-TA was as follows: before the procedure, the cross-sectional abdominal imaging was reviewed; the solid pancreatic mass was then identified and confirmed by EUS. The decision to perform tissue acquisition technique (FNA or FNB) was at the discretion of the attending physicians: either needle type, the standard suction technique (20 mL, negative pressure) or the stylet slow pull technique, with or without fanning method. There were many factors for the decision such as the small tumour size and some endoscopist preferred biopsy needle but aspiration needle was preferred in limited budget patients. The location for TA, transgastric or transduodenal approach, was based on the location of the mass and endoscopists’ decisions. The FNA needle was a 22-gauge EZ-Shot (model number NA-200H-8022; Olympus Medical system, CORP., Japan), whereas the FNB needle was a 22-gauge Franseen needle (Acquire; Boston Scientific, USA). The needle was moved backward and forward within the lesion for at least 15 strokes. ROSE was unavailable for this procedure for an entire period of the study.

The tissue samples were expelled from the needle in a standardized manner. The stylet was firstly introduced at the tip of the needle to expel the tissue samples on the glass slide, followed by flushing the needle with air or normal saline to expel the retained tissue, which called the “touch and smear technique” [18].

The gross tissue specimen was initially evaluated by an endoscopist for adequate tissue sample (white or yellowish tissue at least 2 mm). This procedure is defined as macroscopic onsite evaluation (MOSE). The core tissues were collected in 10% neutral buffered formalin, and the smear glass slide was fixed in 95% alcohol solution for cytology.

The procedure-related data (total procedure time, needle type, location of tissue acquisition, number of needle passes, number of strokes per pass and immediate complication) were recorded into the Endo Smart programme. Finally, glass slides and core tissue samples were sent to the hospital’s Anatomical Pathology Center, where they were reviewed by an experienced cytopathologist.

All tissue samples of eligible patients were reviewed by a single experienced pathologist who was blinded to the procedural detail. The pathological results of ‘positive for adenocarcinoma or adenocarcinoma or carcinoma’ were considered adenocarcinoma. The pathological results of ‘suspicious for malignancy’ were then reviewed by two pathologists for final decision making whether they should be categorized as malignancy or not. The results of a specific diagnosis, such as lymphoma, tuberculosis or neuroendocrine tumour, were also reviewed. However, the definite diagnosis of mass-forming chronic pancreatitis was difficult to attain; it required not only a pathological result indicating fibrosis or chronic inflammation without malignant cell suspicion, but also cross-sectional imaging compatible with chronic pancreatitis and a stable clinical condition without evidence of malignancy on interval cross-sectional imaging after 6 months of follow-up. Meanwhile, the pathological results of unremarkable pancreatic tissue, nonpancreatic elements, atypical pancreatitis or acute pancreatitis were identified as non-diagnostic.

In patients with pancreatic cancer, the disease staging and surgical resectability criteria were applied by The American Joint Committee on Cancer (AJCC) cancer staging manual 8th edition and TNM staging [19].

### Data collection

After eligible patients were identified from the endoscopic centre’s database, we collected the following demographic and clinical characteristics: age; sex; tumour location; CT or MRI results; initial laboratory data; ECOG status; procedural data (EUS findings, needle type, EUS-TA procedure time, technical details and complications following the procedure); pathological report, final diagnosis and disease stage by (AJCC) 8th edition if pancreatic cancer was diagnosed; and diagnosis yield using the hospital’s medical electronic database.

### Statistical analysis

The patients were categorized into EUS-FNA and EUS-FNB groups. The comparisons of continuous variables between the two groups were analysed using Wilcoxon for non-normally distributed data and Student’s *t*-test for normally distributed data, whereas the categorical data were compared using the chi-square test or Fisher’s exact test. A *p*-value of < 0.05 was considered statistically significant. All statistical analyses were performed using the R programme (R foundation for statistical computing, Vienna, Austria).

## Results

During the study period, there were 183 patients who underwent EUS-TA; of those, a total of 151 patients whose EUS-TA was technical successfully performed using 22-G needles were eligible and included in this analysis (88 males; mean age, 62.3 ± 12.8 years), although the study was non-randomized and retrospectively collected, the number of patients in the two groups were similar: 77 in the EUS-FNA group and 74 in EUS-FNB group. Table 1 summarizes the baseline characteristics of the patients in each group. Age, sex, ECOG status and baseline pancreatic mass sizes were not significantly different between the two groups. The majority of patients in this study were 60 years or older but still in the good performance status as > 80% of both groups were ECOG classes 0–1. The mean baseline platelet counts were higher, and the median prothrombin time was shorter in patients in the FNB group; nonetheless, the INR between the two groups was comparable.

**Table 1:** Demographic characteristics of patients in FNA and FNB group

Variables	EUS-FNA (*n* = 77)	EUS-FNB (*n* = 74)	*P* value
Sex (male), *n* (%)	46 (60)	42 (57)	0.710
Age (year)^a^	62.7± 12.8	62.3 ± 12.8	0.671
ECOG status, *n* (%)			0.444
Classes 0–1	66 (85.7)	60 (81.1)	
Classes 2–4	11 (14.3)	14 (18.9)	
Size of solid pancreatic mass, cm^b^	3.5 (2.9, 4.6)	3.8 (3.1, 4.9)	0.289
Initial total bilirubin (mg/dL)^a^	4.7 ± 8.4	6.7 ± 10.6	0.204
Initial platelet count (× 10^3^)^a^	272.8 ±102	318.7 ± 98.8	0.006
Initial haematocrit (%)^a^	36 ± 5.2	35.1 ±5.3	0.309
Initial PT (s)^b^	13.2 (12.4, 14.4)	12.6 (11.6, 13.8)	0.010
Initial INR^b^	1.1 (1.1, 1.2)	1.1 (1.1, 1.2)	0.366

*ECOG *Eastern Cooperative Oncology Group, *PT *prothrombin time, *INR *international normalized ratio

^a^ Data are expressed as mean ± SD

^b^ Data are expressed as median (IQR)

### Solid pancreatic mass characteristics and EUS-TA procedure

As mentioned in Table 1, the median pancreatic mass size in both groups was comparable (3.5 cm vs 3.8 cm in the EUS-FNA and EUS-FNB groups, respectively, *p*=0.289). The solid pancreatic masses were mostly located in the pancreatic head in both groups (FNA, 65%; FNB, 47%). Given that most tumours were in the pancreatic head, the transduodenal approach was used in approximately two-thirds of the patients. Table 2 demonstrates the comparisons of EUS-TA procedural detail between the two groups. The procedure time was significantly shorter in the FNB group (34.7 min) than in the FNA group (41 min, *p* < 0.001). The mean number of needle passes was also significantly different, as the patients in the FNB group required a lower number of needle passes (FNA 3.7 passes vs FNB 2.8 passes, *p* < 0.001).

**Table 2:** Baseline pancreatic mass characteristics and the detail of the EUS-TA procedure between the two groups

Variables	EUS-FNA (*n* = 77)	EUS-FNB (*n* = 74)	*p*-value
Mass location (pancreas), *n* (%)			0.166
Head	50 (64.9)	35 (47.3)	
Uncinate process	6 (7.8)	9 (12.2)	
Body	16 (20.8)	23 (31.1)	
Tail	4 (5.2)	4 (5.4)	
> 1 location	1 (1.3)	3 (4)	
Procedure technique, *n* (%)			0.728
Transgastric approach	25 (32.5)	27 (36.5)	
Transduodenal approach	52 (67.5)	47 (63.5)	
EUS-TA technique, n(%)			
Suction	5 (6.5)	4 (5.4)	1
Stylet slow-pull	15 (19.5)	64 (86.5)	< 0.01
Both suction and stylet slow-pull	57 (74)	6 (8.1)	< 0.01
Procedure time, minutes^a^	41.3 ± 9.2	34.7 ± 9.6	< 0.001
Number of needle passes, *n*^a^	3.7 ± 0.6	2.8 ± 0.6	< 0.001
Number of needle strokes/pass, *n*^a^	18.9 ± 1.9	19.5 ± 2.5	0.079

^a^ Data are expressed as mean ± SD

The EUS-TA tissue sample was initially evaluated by an endoscopist for tissue adequacy (white or yellow tissue at least 2 mm) before being sent to the pathologist. The appropriate tissue sampling was reported by a pathologist; the FNB group had a significantly higher rate of tissue adequacy (100%) than the FNA group (74%) as shown in Table 3. The pathologist also confirmed the definite diagnosis. The diagnostic yield after the first-time tissue sampling was also higher in the FNB group than in the FNA group (94.6% vs 89.6%), but the statistically significant level was not reached. Only one patient in the FNB group experienced an adverse event, of post-procedure mucosal bleeding, which was successfully treated by endoscopic haemostasis.

**Table 3:** Diagnostic performance and complications

Variables	EUS-FNA (*n* = 77)	EUS-FNB (*n* = 74)	*P* value
Adequate tissue reported by a pathologist, *n* (%)	57 (74)	74 (100)	< 0.001
Diagnostic yield, *n* (%)			
Overall	69 (89.6)	70 (94.6)	0.406
In mass size < 4 cm	39/47 (83)	40/43 (93)	0.146
In mass size > 4 cm	30/30 (100)	30/31 (96.8)	1
Diagnosis following EUS-TA, *n* (%)			0.232
Pancreatic adenocarcinoma	40 (51.9)	52 (70.3)	
Pancreatic neuroendocrine tumour	8 (10.4)	5 (6.8)	
Mass forming chronic pancreatitis	10 (13)	3 (4.1)	
Pancreatic lymphoma	4 (5.2)	3 (4.1)	
Pancreatic tuberculosis	2 (2.6)	1 (1.4)	
Other diseases	5 (6.5)	6 (8.1)	
Non-diagnostic	8 (10.4)	4 (5.4)	
Severe adverse event, *n* (%)	0 (0)	1 (0.7)	0.49
Stage of disease in patient with pancreatic cancer (AJCC), *n* (%)	*N* = 40	*N* = 52	0.087
I	1 (2.5)	1 (1.9)	
II	8 (20)	3 (5.8)	
III	21 (52.5)	26 (50)	
IV	10 (25)	22 (42.3)	

The definite diagnoses of solid pancreatic masses after EUS-TA are also shown in Table 3. Malignant mass was the most common aetiology comprising 76.3% of all patients who underwent EUS-TA; 66.3% were pancreatic adenocarcinoma, 5% were pancreatic lymphoma and 5% were metastasis cancer.

Non-malignant tumour, mass-forming chronic pancreatitis and tuberculosis were found in descending order. In patients with pancreatic adenocarcinoma, the cancer staging according to the AJCC 8th criteria is also reported in Table 3.

Interestingly, there were 4 cases of metastasis cancer in the FNB group in which core tissue samples played a major role in aiding the diagnosis; there were one case of metastatic melanoma and three cases of metastasis adenocarcinoma of the lung which could be diagnosed by specific immunohistochemistry.

In subgroup analysis according to the tumour size of not exceeding 4 cm and larger (Table 3), we found that the diagnostic yield was impressively high in both groups when the mass size was > 4 cm, as only 1 out of 61 patients was non-diagnostic. Whereas in patients with mass size < 4 cm, which corresponded with the resectable criteria in pancreatic cancer using AJCC 8th edition, the diagnostic yield in the EUS-FNB group was insignificantly higher than in the EUS-FNA group (93% vs 83%, *p* = 0.146). The location of the pancreatic mass was not associated with the diagnostic yield following EUS-TA.

## Discussion

The pathological diagnosis of solid pancreatic mass has a crucial role in achieving a definite diagnosis and providing proper management, especially in an early stage, which correlates with prognosis in patients with cancer. Adequate tissue sampling might improve the diagnosis and avoid unnecessary surgery in some particular conditions, such as autoimmune pancreatitis, lymphoma and tuberculosis [9, 20, 21]. The EUS-guided core needle biopsy has been designed for larger tissue samples, leading to a better architecture for histological assessment. Moreover, immunohistochemistry staining or genetic testing can be further performed in a case of solid or haematologic malignancies; nonetheless, this staining or testing can be challenging in the tissue obtained from FNA needles [13].

In this study, we evaluated the diagnostic yield of EUS-TA by FNA versus FNB using the same size 22-G needles in a centre where ROSE is not available in patients with solid pancreatic mass lesion detect by cross-sectional imaging. We found that EUS-TA using the Franseen (Acquire) FNB needle had a significantly higher rate of tissue adequacy when compared with FNA needle (100% vs 74%, *p* < 0.001), and the diagnostic yield was also higher in FNB than in FNA needle (94.6% vs. 89.6%); however, the statistically significant level was not reached. EUS-FNB using the 3rd-generation needle in our study was also associated with a shorter procedure time and fewer numbers of needle passes when compared with FNA significantly.

The ESGE guidelines recommended either 25- or 22-gauge needle sizes for tissue sampling of the solid pancreatic mass regardless of the needle type [8]. In a previous meta-analysis, the diagnostic sensitivity and specificity in both 25- and 22-gauge FNA needles were comparable [16, 17, 22]. And for the needle types, fork-tip shape and Franseen needle used in FNB was not significantly different in diagnostic accuracy [23], as well as the needle types used in FNA; there were no significant differences in both diagnosis accuracy or sampling adequacy [24]. In earlier studies, the diagnostic yields of EUS-FNB using the first and second-generation needles were not superior than those using EUS-FNA [10–12]. But the data regarding the use of the 3rd-generation FNB needles in comparison with FNA are still limited.

One of the factors reported to be associated with a better diagnostic accuracy was ROSE (odds ratio for better accuracy of 2.6; 95%CI 1.41–4.79) [25]. In previous reports, the diagnostic yields of EUS-TA without ROSE by FNA were 70–82%, whereas those by FNB were 70–89%, respectively [10, 14, 25, 26]. However, EUS-TA with ROSE increased the diagnostic performance to 77.5–80% in FNA and 87–90% in FNB, respectively [16, 27]. In centres where ROSE was available, there was a study that showed that the diagnostic yields between FNB and FNA using the same size needle were comparable, but the number of needle passes was fewer in the FNB group [12, 28, 29]. Therefore, ROSE appeared to be substantially helpful in improving diagnostic yield in EUS-TA procedures. In centres with ROSE available, the benefit of EUS-FNB over EUS-FNA was not the superiority in diagnostic yield but the reduction in the number of needle passes and total procedure time [17].

Nonetheless, in many limited-resource countries, onsite cytopathologists are not available, and ROSE is inevitably impractical, including in Thailand. We conducted this study to evaluate whether the 3rd-generation FNB needle provided a better diagnostic yield than FNA when the tissue acquisition was performed without ROSE. Our study illustrated that, in centre without onsite cytopathologist, the 3rd-generation Franseen FNB needle offered a significantly higher tissue adequacy for histopathological assessment and also achieved a higher diagnostic yield than the FNA needles, although the statistically significant level was not reached. The results of our study were also concordant with prior studies in terms of shorter procedure time and fewer needle-passed in the FNB group compared to the FNA group [15]. Moreover, we observed a higher diagnostic yield of FNB than in FNA groups in patients with pancreatic mass size not exceeding 4 cm (93% vs 83%, *p* = 0.146). Although it was statistically insignificant, it might be of clinical importance as the tumour size of 4 cm or smaller was one of the major resectable criteria in patients with pancreatic cancer.

The very high diagnostic yields of EUS-FNB in our study, over 90% in both mass sizes do of not exceeding 4 cm and over, might be explainable by the use of the 3rd-generation biopsy needle which was designed for harvesting a larger size of core tissue specimen, making it more feasible to evaluate tissue adequacy macroscopically onsite by endoscopists (MOSE). In addition, the endoscopists’ level of experience in our study may also play a role, as all endosonographers usually performed EUS-TA more than 50 cases/year during the entire study period.

Our study highlighted the benefits of EUS-FNB using 3rd-generation needles in solid pancreatic lesions over EUS-FNA in centres where ROSE was not available. Not only a lower number of needle passes (2.8 vs 3.7, *p* < 0.001) or saving the total procedure time (34.7 vs 41 min, *p* < 0.001), EUS-FNB also provided better diagnostic performance than FNA, especially in tumour size of ≤ 4 cm, while the adverse events were comparable between the groups the same as in previous reports. The complication rate of EUS-FNA is approximately 1–2%, and the adverse event was comparable between the EUS-FNB and the EUS-FNA [30, 31]. The previous report concerned in needle tract seeding followed tissue acquisition but this was not shown in our report [32]. Although the cost of FNB needle was higher than that of FNA, a higher proportion of patients could have a definite diagnosis which might promote an early treatment, especially in those with malignancy, whereas in non-diagnostic tissue specimens, patients needed to undergo a repeat EUS-TA, which might cause delayed diagnosis or higher overall procedural cost.

This study was conducted in a tertiary university hospital and included patients with solid pancreatic mass initially assessed by cross-sectional imaging; the FNB and FNA groups had similar baseline characteristics. ROSE was unavailable in our centre, and this might be a pioneer study of a pragmatic setting of unavailable ROSE in many centres worldwide.

Moreover, our study demonstrates the benefits of FNB in various aetiologies, such as tuberculosis, lymphoma and metastatic cancer which required core tissue or culture for a definite diagnosis. The limitation of this study is the retrospective design, and the subjects were commonly in the unresectable stage referred for tissue diagnosis before initiation of systemic chemotherapy. Although a better diagnostic yield was also observed in patients whose mass size ≤ 4 cm undergoing EUS-FNB, it is important to note that some patients who were considered to be resectable pancreatic cancer from cross-sectional imaging might proceed to curative surgery without pre-operative EUS-TA. Further studies in patients with solid pancreatic lesion size ≤ 4 cm are needed to confirm the benefit of EUS-FNB in comparison with EUS-FNA.

## Conclusion

The diagnostic yield of solid pancreatic mass was higher in FNB using the Franseen needle than FNA using the conventional FNA needle in a centre where ROSE is unavailable, without serious adverse event. In addition, FNB had fewer needle passes and shorter total procedure time.

## Data Availability

Due to ethical restrictions, the dataset related to the current study is available upon request to the corresponding author.

## Abbreviations

EUS: Endoscopic ultrasonography
TA: Tissue acquisition
FNA: Fine-needle aspiration
FNB: Fine-needle biopsy
ROSE: Rapid onsite evaluation
MOSE: Macroscopic onsite evaluation
ECOG: Eastern Cooperative Oncology Group
MRI: Magnetic resonance imaging
CT scan: Computerized tomography scan
